# Determinants of Childhood Obesity in Representative Sample of Children in North East of Iran

**DOI:** 10.1155/2012/875163

**Published:** 2012-07-15

**Authors:** Fereshteh Baygi, Ahmad Reza Dorosty, Roya Kelishadi, Mostafa Qorbani, Hamid Asayesh, Morteza Mansourian, Kamal Mirkarimi

**Affiliations:** ^1^Endocrinology and Metabolism Research Center, Tehran University of Medical Sciences, Tehran 1411413137, Iran; ^2^Department of Nutrition and Biochemistry, Tehran University of Medical Sciences, Tehran, Iran; ^3^Pediatrics Department, Faculty of Medicine and Child Growth and Development Research Center, Isfahan University of Medical Sciences, Isfahan, Iran; ^4^Department of Epidemiology and Biostatistics, Tehran University of Medical Sciences, Tehran 1411413137, Iran; ^5^Faculty of Paramedical, Qom University of Medical Sciences, Qom, Iran; ^6^Department of Health Education and Promotion, Tehran University of Medical Sciences, Tehran, Iran; ^7^Department of Health Education and Promotion, Golestan University of Medical Sciences, Gorgan, Iran

## Abstract

Childhood obesity has become, a global public health problem, and epidemiological studies are important to identify its determinants in different populations. This study aimed to investigate factors associated with obesity in a representative sample of children in Neishabour, Iran. This study was conducted among 1500 randomly selected 6–12-year-old students from urban areas of Neishabour, northeast of Iran. Then, through a case-control study, 114 obese (BMI ≥ 95th percentile of Iranian reference) children were selected as the case group and were compared with 102 controls (15th ≤ BMI < 85th percentile). Factors suggested to be associated with weight status were investigated, for example, parental obesity, child physical activity levels, socio-economic status (SES), and so forth. The analysis was conducted using univariate and multivariate logistic regression (MLR) in SPSS version 16. In univariate logistic regression model, birth weight, birth order, family extension, TV watching, sleep duration, physical activity, parents' job, parents' education, parental obesity history, and SES were significantly associated with children's obesity. After MLR analysis, physical activity and parental obesity history remained statistically significant in the model. Our findings showed that physical activity and parental obesity history are the most important determinants for childhood obesity in our population. This finding should be considered in implementation of preventive interventions.

## 1. Introduction

Obesity levels are increasing rapidly in children and youngsters of developed and developing countries [1, 2]. In 1998, the World Health Organization project monitoring of cardiovascular diseases (MONICA) showed that Iran is one of the seven countries with the highest prevalence of childhood obesity [3]. Overweight and obesity among Iranian children are becoming a major public health problem [4]. It is predicted that, by 2020, more than 60% of diseases and their related mortality and morbidity in the developing countries will be due to noncommunicable diseases, for many of which obesity is a potential risk factor [5].

Obesity is a multifactorial consequence. In addition to genetic, metabolic, socioeconomic, and cultural factors, life style habits as unhealthy diet, low physical activity levels, weight and order of birth and other factors like history of breast feeding, as well as the age and type of complementary food are among factors affecting obesity [6]. Therefore, this study was designed to identify probable factors that might affect obesity in a group of Iranian children.

## 2. Materials and Methods 

### 2.1. Study Design

In this study, 1500 6–12-year-old students were selected via two-stage sampling method from urban areas of Neishabour. Neishabour is a city in the Razavi Khorasan province in northeastern Iran and had a population of 205972 people. On the first stage, 60 primary schools, both public and private, were chosen, and, on the second stage, in each cluster, school children were selected randomly from the class attendance register. It was approved by the ethics committee of Tehran University of Medical Sciences (TUMS). Written informed consent was obtained from parents and oral assent from students.

Then, through a case-control study, 14 obese (body mass index (BMI) ≥ 95th percentile of Iranian reference) children were selected as the case group, and the first nonobese student (15th ≤ BMI < 85th percentile) examined exactly after each obese student, and who was matched by age and sex, was selected as the control. Overall, 102 students were included in the control group.

### 2.2. Anthropometric Profile

We used the Iranian reference for BMI percentiles [7]. Physical examination was conducted by a trained team of health professionals using calibrated instruments. Height was measured by a stadiometer (Seca, Germany) in a standing position with bare feet (precision 0.5 cm), and body weight was determined with subjects wearing light clothes and no shoes or socks, using an electronic balance. BMI was calculated as weight (kg) divided by height squared (m^2^).

### 2.3. Data Collection

Data were collected by questionnaire via interview with mothers. Interviews were performed by trained health professionals. The questionnaire included mother-reported information about her child regarding the age, sex, birth weight, birth order, number of family household members, duration of breast feeding, age onset of complementary food, TV watching, playing electronic devices, sleep duration, father age, mother age, mother weight, economic status, and parental obesity history. The economic status of family was assessed by having some equipment such as color TV, refrigerators, washing machine, video, computer, video CD, and accessibility to car and private home. In data analysis, economic status was defined as low, moderate, and good based on an average score 3, 4–6, and more than 7, respectively. Physical activity score was evaluated using the modified Beacke et al. questionnaire that was asked from the pupils [8]. 

### 2.4. Statistical Analysis

Data were analyzed using the SPSS software version 16.0 (SPSS Inc., Chicago, IL, USA). Quantitative variables are expressed as mean ± standard deviation (SD) and categorical data as percentage. We computed the crude odds ratio (OR) by using logistic regression test to establish the degree of association between various risk factors and childhood obesity. Multiple logistic regression (MLR) model was fitted to data to adjust for the presence of confounding factors. All variables with *P* value < 0.1 in the univariate analysis were presented to the multiple model by using stepwise method. The results of logistic regression analysis are presented by OR and 95% confidence interval. A *P* value of less than 0.05 was considered as statistically significant.

## 3. Results

### 3.1. Demographic Characteristics

 Overall, 216 children consisting of 114 obese and 102 nonobese students were assessed. The mean (SD) of age, weight, height, and BMI of the students were 8.6 ± 1.5 years, 33.4 ± 9.9 kg, 132.3 ± 9.5 cm, and 18.7 ± 3.8 kg/m^2^, respectively.

### 3.2. Determinant Factors

The crude OR_S_ (univariate analysis) for birth weight, birth order, family extension, duration of breast feeding, age onset of complementary food, TV watching, playing electronic devices, sleep duration, physical activity score, father age, mother age, mother BMI as quantitative variables are shown in Table 1. The estimated crude ORs for birth weight, birth order, family extension, TV watching, playing electronic devices, sleep duration, physical activity score, father age, mother age, and mother BMI were statistically significant. 

 Associations between qualitative variables and obesity determined by univariate logistic regression model are shown in Table 2. The estimated crude ORs for father job, father education, mother job, mother education, economic status, and parental obesity history were statistically significant. After MLR analysis, at final step, physical activity and parental obesity history remained significant in the model (Table 3).

## 4. Discussion

This study has demonstrated that parental obesity history and physical activity were the strongest determinants of childhood obesity. These findings are in agreement with some investigations, showing that low physical activity and parental obesity to be the predictors of youngsters obesity [9–11].

The result of Veugelers and Fitzgerald's study showed that, as in other studies, normal-weight children were more physically active and engaged less in sedentary activities [12].

In our study, there was no significant association between mothers' BMI and children obesity, but Sekine and his collogues found that mother's obesity was associated with children's BMI. In addition to genetic susceptibility, this may reflect environmental factors influencing children's body composition because children's food habits and preferences are usually shaped more by mothers than fathers [11]. 

The controversy between our result and other studies may be because of some factors related to family composition that we did not ask in our study and it may confound our conclusion. Our findings are also consistent with a previous study, which showed that a positive family history of being overweight is one of the most important indicators of the genetic risk for obesity and being overweight [13].

We did not document any relationship between SES and obesity, but, in some other studies, investigators observed a gradient whereby children by low SES were more likely to be overweight or obese [12, 14]. This contradiction between our study and others may be related to the homogenous SES of families studied and also because differences in the SES indicators were used. For instance, we asked some questions about having some equipment at home for assessing family income, but other researchers considered direct family income for evaluating economic status [15].

In present study, we did not find any association between the sleep duration and obesity. Some studies demonstrated an inverse linear relationship between sleep duration and both mean BMI and obesity [16–18]. Researchers believe that there is association between the secretion of growth hormone and sleeping early at night. Going to sleep early at night increases the secretion of the growth hormone, which activates lipolysis in fat tissues, and obviously sleeping late increases the rate of obesity [19].

But, in our study, no significant association was observed between sleep duration at night and obesity. Maybe in current study, individuals of the case group went to bed later than the controls, and, in order to compensate for the lack of sleep, they slept more during the day instead of being more physically active or exercising.

Our study revealed no significant association between obesity and birth order. It may be related to increasing the knowledge of parents and paying more attention to the nutrition and health care of their children in both case and control groups.

In a meta-analysis carried out by Harder, the duration of breastfeeding was inversely and linearly associated with the risk of overweight. The risk of overweight was reduced by 4 percent for each month of breastfeeding [20]. Investigators think the protective effect of breast milk against obesity is attributed to its special components. Long-chain polyunsaturated fatty acid in breast milk may reduce the risk of obesity in the adulthood. High concentration of these fatty acids in the brain inhibits the production of cytokines and increases insulin receptors in several tissues which improves insulin, and other neurotransmitter functions. The dietary intake is regulated by complex interactions of some neurotransmitters, insulin and its receptors in the brain indicate the necessity of taking these fatty acids in the first year of life [20]. But, in our study, we find no association between obesity and duration of breast feeding and age of beginning complementary foods.

## 5. Limitation

This study had some limitations, which may have influenced the findings. First, the SES was measured indirectly by asking some relevant questions. Second, family composition was not assessed in this study, and it may be a confounder for some parts of our results. Despite these limitations, this study provides some data on childhood obesity risk factors.

## 6. Conclusion

Finally, we suggested that a high priority has been given to research strategies to prevent the development of childhood obesity. Also, based on our findings, more physical education classes and providing healthy places for more extracurricular physical activity are strongly recommended, and family education for preventive public health actions should be targeted.

## Figures and Tables

**Table 1:** Association between quantitative variables and obesity in univariate logistic regression model.

Variables	Cases (*n* = 114)	Control (*n* = 102)	Crude OR	95% CI
	(mean ± SD)	(mean ± SD)		
Birth weight (gr)	3900. 90 ± 846.20	2837.10 ± 671.30	1.00	1.00–1.00
Birth order (n)	1.90 ± 1.20	5.09 ± 13.05	0.62	0.50–0.76
Family extension (n)	4.40 ± 0.97	5.10 ± 1.60	0.63	0.49–0.80
Duration of breast feeding (month)	22.02 ± 9.80	23.76 ± 14.33	0.99	0.97–1.11
Age-onset of complementary food (month)	8.77 ± 17.56	9.63 ± 15.63	1.00	0.98–1.01
TV watching, playing electronic devices (hour)	5.42 ± 1.99	2.77 ± 1.26	2.33	1.87–2.90
Sleep duration (hour)	10.40 ± 0.91	9.76 ± 0.89	2.24	1.60–3.15
Physical activity (score)	2.30 ± 0.37	3.02 ± 0.39	0.01	0.01–0.04
Father age (year)	39.57 ± 5.37	41.71 ± 6.27	0.94	0.90–0.99
Mother age (year)	35.51 ± 5.84	37.50 ± 6.88	0.95	0.91–0.99
Mother BMI (kg/m^2^)	26.98 ± 3.99	22.77 ± 3.81	1.40	1.25–1.55

**Table 2:** Association between qualitative variables and obesity in univariate logistic regression model.

Variables	Cases (*n *= 114)	Control (*n *= 102)	Crude OR	95% CI
	*N* (%)	*N* (%)		
Father job				
Jobless	1 (0.90)	4 (4.40)	Ref	
Worker	17 (15.00)	31 (31.60)	2.19	0.23–21.23
Employee	34 (30.10)	40 (40.80)	3.40	0.36–31.89
Other	61 (54.00)	23 (23.50)	10.60	1.13–99.96
Father education				
Illiterate and primary school	23 (20.40)	36 (36.70)	Ref	
Guidance school	16 (14.20)	29 (29.60)	0.86	0.39–1.92
Diploma	24 (21.20)	21 (21.40)	1.78	0.816–3.92
University degrees	50 (44.20)	12 (12.20)	6.52	2.87–14.79
Mother job				
Housewife	55 (48.20)	80 (78.40)	Ref	
Employee	59 (51.80)	22 (21.60)	3.90	2.14–7.09
Mother education				
Illiterate and primary school	35 (30.70)	49 (48.00)	Ref	
Guidance school	12 (10.50)	33 (32.40)	0.51	0.23–1.12
Diploma	12 (10.50)	14 (13.70)	1.20	0.50–2.90
University degrees	55 (48.20)	6 (5.90)	12.80	4.97–33.10
Economic status				
Weak	18 (15.80)	72 (63.20)	Ref	
Moderate	72 (63.20)	57 (55.90)	3.01	1.57–5.78
Good	24 (21.10)	2 (2.00)	28.66	6.12–134.20
Family history of obesity				
Without obesity history	7 (6.10)	76 (74.50)	Ref	
Father side obesity history	24 (21.10)	11 (10.80)	23.68	8.26–67.89
Mother side obesity history	5 (4.40)	13 (12.70)	4.17	1.15–15.16
Both of them	78 (68.40)	2 (2.00)	423.42	85.24–2010.00

**Table 3:** Association between independent variables and obesity in multiple logistic regression model (last step).

Variables	Adjusted OR	^ ∗^95% CI
Physical activity (score)	0.23	0.12-0.43
Family history of obesity		
Without obesity history	Ref	
Father side obesity history	47.41	9.87-227.60
Mother side obesity history	2.36	0.39-14.32
Both of them	547.58	45.26-6062.00

*Adjusted for birth weight, birth order, family extension, TV watching, playing electronic devices, sleep duration, physical activity score, father age, mother age, mother BMI, father job, father education, mother job, mother education, economic status, and parental obesity history.

## References

[1] Li AY, Kaye B, Rosemary C, Liang K, Stephen M (2008). Parental obesity as a predictor of childhood overweight/obesity in Australian migrant children. *Obesity Research & Clinical Practice*.

[2] Strauss RS, Knight J (1999). Influence of the home environment on the development of obesity in children. *Pediatrics*.

[3] Kelishadi R, Pour MH, Sarraf-Zadegan N (2003). Obesity and associated modifiable environmental factors in Iranian adolescents: Isfahan healthy heart program—heart health promotion from childhood. *Pediatrics International*.

[4] Dorosty AR, Siassi F, Reilly JJ (2002). Obesity in Iranian children. *Archives of Disease in Childhood*.

[5] Caballero B (2001). Obesity in developing countries: biological and ecological factors. *Journal of Nutrition*.

[6] Speiser PW, Rudolf MCJ, Anhalt H (2005). Consensus statement: childhood obesity. *The Journal of Clinical Endocrinology & Metabolism*.

[7] Hosseini M, Carpenter RG, Mohammad K, Jones ME (1999). Standardized percentile curves of body mass index of Iranian children compared to the US population reference. *International Journal of Obesity and Related Metabolic Disorders*.

[8] Baecke JA, Burema J, Frijters JE (1982). A short questionnaire for the measurement of habitual physical activity in epidemiological studies. *American Journal of Clinical Nutrition*.

[9] Moore LL, Di Gao AS, Bradlee ML (2003). Does early physical activity predict body fat change throughout childhood?. *Preventive Medicine*.

[10] Danielzik S, Langnäse K, Mast M, Spethmann C, Müller MJ (2002). Impact of parental BMI on the manifestation of overweight 5-7 year old children. *European Journal of Nutrition*.

[11] Sekine M, Yamagami T, Hamanishi S (2002). Parental obesity, lifestyle factors and obesity in preschool children: results of the toyama birth cohort study. *Journal of Epidemiology*.

[12] Veugelers PJ, Fitzgerald AL (2005). Prevalence of and risk factors for childhood overweight and obesity. *Canadian Medical Association Journal*.

[13] Kries RV, Berthold K, Sauerwaldet T (1999). Breast feeding and obesity: cross sectional study. *British Medical Journal*.

[14] Power C, Graham H, Dueet P (2005). The contribution of childhood and adult socioeconomic position to adult obesity and smoking behavior: an international comparison. *International Journal of Epidemiology*.

[15] Wang Y (2001). Cross-national comparison of childhood obesity: the epidemic and the relationship between obesity and socioeconomic status. *International Journal of Epidemiology*.

[16] Park S (2011). Association between short sleep duration and obesity among South Korean adolescents. *Western Journal of Nursing Research*.

[17] Hart CN, Jelalian E (2008). Shortened sleep duration is associated with pediatric overweight. *Behavioral Sleep Medicine*.

[18] Marshall NS, Glozier N, Grunstein RR (2008). Is sleep duration related to obesity? A critical review of the epidemiological evidence. *Sleep Medicine Reviews*.

[19] Gupta NK, Mueller WH, Chan WW, Meininger JC (2002). Is obesity associated with poor sleep quality in adolescents?. *American Journal of Human Biology*.

[20] Das UN (2001). Is obesity an inflammatory condition?. *Nutrition*.

